# MicroRNA of Epithelial to Mesenchymal Transition in Fuchs’ Endothelial Corneal Dystrophy

**DOI:** 10.3390/genes13101711

**Published:** 2022-09-23

**Authors:** Spela Stunf Pukl

**Affiliations:** 1 Medical Faculty, University of Ljubljana, 1000 Ljubljana, Slovenia; spela.stunf@siol.net; Tel.: +386-41-382-487; 2 University Eye Hospital, University Clinical Centre Ljubljana, 1000 Ljubljana, Slovenia

**Keywords:** epithelial mesenchymal transition, Fuchs’ endothelial corneal dystrophy, FECD, miRNA, microRNA

## Abstract

Aim: a review of miRNA expression connected to epithelial mesenchymal transition studies in Fuchs’ endothelial corneal dystrophy (FECD). Methods: literature search strategy—PubMed central database, using “miRNA” or “microRNA” and “epithelial mesenchymal transition” or “EMT” and “Fuchs’ endothelial corneal dystrophy” or “FECD” as keywords. Experimental or clinical studies on humans published in English regarding miRNA profiles of epithelial mesenchymal transition in Fuchs’ endothelial corneal dystrophy published between 2009 and 2022 were included. Conclusion: The publications regarding the miRNA profiles of epithelial mesenchymal transition in Fuchs’ endothelial corneal dystrophy are scarce but provide some valuable information about the potential biomarkers differentiating aging changes from early disease stages characterized by epithelial mesenchymal transition. In the corneal tissue of FECD patients, miRNA-184 seed-region mutation as well as unidirectional downregulation of total miRNA expression led by the miRNA-29 were demonstrated. For early diagnostics the miRNA of epithelial mesenchymal transition in aqueous humor should be analyzed and used as biomarkers.

## 1. Introduction

**Figure 1:**
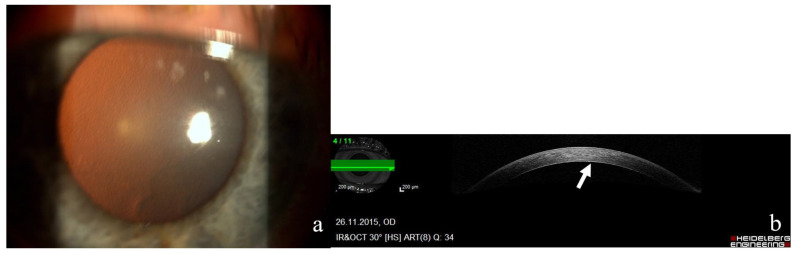
(**a**) Photograph of the right cornea of a 65-year-old female patient with moderate-stage Fuchs’ endothelial corneal dystrophy and characteristic endothelial guttae. (**b**) Anterior-segment optical-coherence tomograph from Heidelberg Spectralis of the same patient’s right cornea. Fine endothelial changes (arrow), corneal thickness is within normal limits—575 mm.

Fuchs’ endothelial corneal dystrophy (FECD) is a corneal disease affecting females three times more often than males. Early-onset FECD manifests in the third decade and late-onset in the fifth decade of life. Basic pathology includes accelerated loss and dysfunction of corneal endothelial cells, epithelial mesenchymal transition of endothelial cells, and abnormal extracellular matrix (ECM) and its accumulation, resulting in thickening of the Descemet membrane and formation of focal-basement membrane excrescences called guttae (Figure 1) [1].

**Figure 2:**
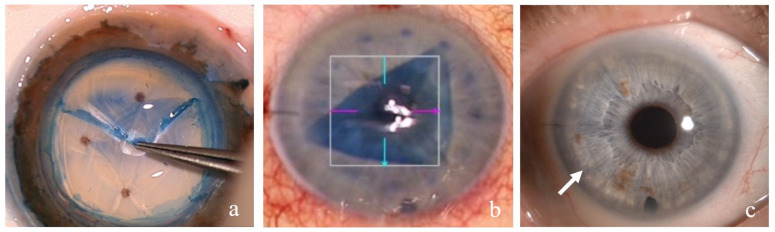
Endothelial corneal transplantation DMEK—Descemet membrane endothelial keratoplasty: (**a**) photograph of the preparation of the DMEK transplant—the Descemet membrane is stained with trypan blue and detached by a gentle forceps pull at the periphery; (**b**) intraoperative view of the anterior chamber with a partially bent trypan blue-stained DMEK transplant in the center of the anterior chamber; (**c**) photograph of the anterior chamber in a 67-year-old female patient 1 month after DMEK—the cornea is clear and transparent, and the peripheral border of the DMEK transplant can be observed (arrow).

FECD starts in the central cornea. The early stages of the disease cause minor visual impairment and can also be asymptomatic. The clinical picture is very similar to aging changes. The diagnosis of FECD can be difficult to establish and is based on the disease progression. On the other hand, moderate FECD causes diurnal changes of corneal edema with significant visual blurring. Advanced FECD causes permanent corneal edema and severe visual deterioration. Treatment is based on restoration of the endothelial pump function. Severe FECD can, to date, only be treated with replacement of the corneal endothelial cells via endothelial transplantation (Figure 2) [2,3].

Early and moderate FECD, where the extent of endothelium changes is still limited to the central 3–4 mm, can be treated without transplant. In the last decade, more and more clinical studies have reported successful treatment with excellent visual outcomes of moderate FECD with removal only of the affected central endothelium (DSO—Descemet stripping only, DWEK—Descemet without endothelial keratoplasty). It is based on the patient’s own healthy peripheral corneal endothelial-cell migration [4,5,6,7,8]. Treatment of FECD without corneal transplantation is an elegant way of avoiding the possible consequences of tissue transplantation (inflammation in the anterior chamber, transplant-tissue rejection, life-long immunomodulatory treatment, etc.) as well as bypassing the constant shortage of corneal tissue in many European regions. However, the migration of the endothelium needs time, which causes prolonged central corneal edema with visual blurring in patients with early-stage FECD and few preoperative complaints. Patients and clinicians could benefit from a biomarker that unambiguously proves the disease in its early stage and justifies early surgical intervention.

Histological changes in FECD with epithelial–mesenchymal transition of endothelial cells with advancing of the disease result in the loss of endothelial cells’ pump function, corneal edema, and opacification [1]. Normally, the Descemet membrane consists of anterior banded and posterior non-bended layer. The anterior has a constant 3 μm thickness after birth, whereas the posterior continuously increases in thickness through the lifespan, from 3 μm at 20 years old to 10 μm at 80 years old [9]. In FECD the Descemet membrane thickens beyond age-related expectations, and the posterior non-banded layer is attenuated or missing. There are additional subendothelial deposits of ECM as subendothelial posterior collagenous fibrillar layers and in the form of excrescences of Descemet membrane called guttae (Figure 1) [10,11,12]. Different pathogenetic mechanisms underlying FECD were suggested and proved in the literature—endoplasmic-reticulum stress, followed by activated cell-protective signaling events called the unfolded protein response, and oxidative stress [13,14]—and the understanding of the genetic, epigenetic, and molecular mechanisms of FECD is still evolving.

The International Classification of Corneal Dystrophies categorizes FECD into two different types: early-onset FECD and late-onset FECD. Early-onset FECD, which begins in the first decade, has been mapped to single genetic loci ascribed to mutations in the collagen type VIII α 2 chain (*COL8A2*, MIM 12052) [15,16,17]. The pathophysiology of the far more frequent late-onset FECD, manifesting around the fifth decade, remains unknown. Genetic predisposition is a risk factor [17,18,19,20,21,22,23]. In fact, it has been linked to a variety of different genetic and environmental factors.

As in the other complex diseases, genetic susceptibility for the disease depends on different genetic variants, acting together or separately. Among them, variants in two genes have been consistently associated with FECD: (1) variants of the α 2 subunit of *COL8A2* [16,24] and (2) variants in transcription factor 4 (*TCF4*) [25,26,27,28,29].

*COL8A2* was found to rarely be mutated [30]. However, some of these mutations, such as the missense mutations Leu450Trp and Gln455Lys, cause highly penetrant, early-onset forms of the disease, which is associated with thickening of the Descemet membrane and subsequent increase in central corneal thickness [16,25]. Some other genetic variants in *COL8A2*, on the other hand, were connected to thin cornea in Caucasians and Asians. Thinning of the cornea was also connected to loss of *COL8A2* in animal studies [31,32,33,34,35,36].

Other genetic mutations at different positions, without a single causative gene mutation, have also been described as being associated with FECD:

- Mutation in solute carrier family 4 member 11 (*SLC4A11*, MIM 610206) [37,38],
- Mutations in transcription factor 8 gene (*TCF8*, MIM 189909) [39],
- Mutations in transcription factor 4 gene (*TCF4*, MIM 602272) [27],
- Mutations in lipoxygenase homology domains 1 (*LOXHD1*, MIM 613072) [40], and
- Mutations in ATP/GTP binding protein like 1 (*AGBL1*, MIM 615496) [41].

Cornea is a tissue that, due to its light refractive function and external location, is constantly exposed to environmental factors. Thus, the influence of epigenetic mechanisms might be more important than the genetic mutations, especially for a disease that has a late-in-life manifestation. The epigenetic mechanisms provide a mechanistic link between environmental risk factors and the etiology of diseases [42]. The ECM changes in FECD and dysfunctional cells result from transition of the epithelial phenotype to mesenchymal. In the early stages, a sole clinical exam of the cornea cannot differentiate between aging and disease. The clinical picture of early FECD and aging changes to the endothelium with few guttae is very similar. However, the progressing nature of corneal opacification due to epithelial-to-mesenchymal transition (EMT) of endothelial cells and loss of their primary pump function is only characteristic of FECD. Yet, the modern treatment approaches focus on early diagnosis and action—removal of the diseased central part of the Descemet and endothelium without transplantation.

There were several reports recently in which an extensive impact of miRNAs in cell response to different stress conditions and diseases was demonstrated. Stress-dependent regulation can involve upregulation or downregulation of miRNA expression and lead to downstream-signaling effects on mRNA targets. How the two are connected through the epigenetic mechanisms of miRNAs is the question behind the following literature search.

Hypothesizing that miRNAs are differentially regulated through the environmental influences, causing cellular and ECM changes and resulting in FECD pathogenesis, a systematic review of the literature was generated in search of a biomarker of EMT for early recognition of FECD in stages still treatable without corneal transplant.

## 2. Materials and Methods

A literature search for publications in English between 2009 and 2022 was performed in the PubMed central database, using “miRNA” or “microRNA” and “epithelial mesenchymal transition” or “EMT and “Fuchs’ endothelial corneal dystrophy” or “FECD” as keywords. Clinical and experimental human studies were critically reviewed.

## 3. Results

### 3.1. MicroRNA

MicroRNAs (miRNAs)—evolutionarily conserved, small, 20 to 24 nucleotides long—and noncoding RNAs have recently attracted attention, as they regulate more than 60% of protein-coding genes, with important function in cellular proliferation, differentiation, and cell death [43]. MicroRNAs bind to messenger RNA (mRNA), causing RNA silencing and suppressing gene expression [44]. They do so by base pairing to their target sites with complementary sequence in the 3′ untranslated region (UTR). Their 5′ seed region (nt 2–7) is usually complementary to the highly conserved regions in 3′ UTRs of mRNAs [45]. Base pairing of five nucleotides in line is usually sufficient for a corresponding miRNA–mRNA interaction [43]. The base pairing is followed by the formation of an induced silencing complex (RISC). The mRNA is next divided into two parts, weakened by the shortening of its poly(A) tail or inefficiently translated into proteins [46]. Because miRNAs can bind to mRNAs using only some of their nucleotides, they can bind multiple mRNA sequences and thus target numerous genes. An individual miRNA may have hundreds of different mRNA targets. For inhibition of a single mRNA, usually many individual miRNAs are required [43,46]. miRNAs weaken or split mRNA or suppress its translation and therefore control eukaryotic gene expression at the posttranscriptional stage [45].

miRNAs were suggested as potential biomarkers of different diseases, including certain corneal diseases, and were also proposed as possible treatment targets [47,48].

### 3.2. Epithelial-to-Mesenchymal Transition and Fuchs’ Endothelial Corneal Dystrophy

EMT is one of the key embryological mechanisms, and involves epithelial cells losing their polarity and obtaining mesenchymal properties. EMT also participates in tissue repair; inflammation; fibrosis, including fibrosis of the cornea after trauma or infection; and tumor metastasis [48,49].

In late stages of FECD both cellular and ECM changes can be found. The endothelial cells are morphologically changed, as is their production of ECM. In fact, multiple EMT-related genes were proven to be implicated in FECD [50]. Cytokine analysis of aqueous humor, which bathes the endothelium, gives insight into the pathological processes of endothelial cells. There are different proteins found in aqueous humor that cause corneal endothelial cells to undergo a change from hexagonal to fibroblast-like in the settings of EMT: (1) fibroblast growth factor 2 (FGF-2) [51,52], (2) transforming growth factor–β (TGF-β) [53,54], (3) monocyte chemoattractant protein-1 (MCP-1) [55], (4) interleukin 1 β (IL-1β) [56], and (5) tumor necrosis factor α (TNF-α) [56,57].

In vitro, TGF-β stimulation of corneal endothelial cells induced their transformation to a fibroblast-like phenotype, with higher production of ECM proteins, such as collagen I and IV and fibronectin [58]. Similarly, fibroblast-like cells and collagen I, collagen IV, and fibronectin accumulation was found in late-stage FECD specimens [13,59,60]. TGF-β 2 and TGF-β 3 in aqueous humor are particular to FECD. After cataract surgery, there is a switch toward TGF-β 1, which is a classical player in the wound-healing processes [61,62,63,64].

Actually, the EMT-inducing genes zinc-finger E-box-binding homeobox 1 (ZEB1) and snail-family transcriptor repressor 1 (SNAI 1) are excessive in corneal endothelial cells in FECD. This in turn renders endothelial cells more responsive to TGF-β from the aqueous humor and results in excessive production of ECM proteins, such as type-I collagen and fibronectin [65,66,67]. *ZEB1* encodes the two-handed zinc-finger homeodomain transcription factor, which is a booster or suppressor of transcription [68]. Missense mutations in *ZEB1* have been reported in FECD (MIM #613270) [39,69,70]. Genetic variation in the transcription factor 4 gene (TCF4; MIM602272) has been associated with the development of FECD in a potential mechanism involving altered *ZEB1* expression [25].

ZEB1 directly regulates several genes related to EMT [69,71]. Therefore, ZEB1 missense mutation in FECD causes its reduced expression and dysregulation of α-type-IV collagens—namely, COL4A3 expression is four- to five-fold higher than in the normal corneal endothelium [69,72]. ZEB1 binding sites are also in the promoter sites of COL8A2 and the basement collagen genes (COL4A1, COL4A2, COL4A3, COL4A5, and COL4A6). In case of the missense mutation in ZEB1 (c.1920G > T;p.Gln640His) COL4A1, COL4A2, and COL4A3 expression are markedly reduced in corneal keratocytes [73]. Disruption of COL8A1 and COL8A2 in a knockout mouse model resulted in corneal thinning in the form of keratoglobus [38]. Missense mutations in COL8A2 resulted in posterior polymorphous corneal dystrophy (PPCD) and FECD, although no pathologic variants have been reported in keratoconus. It has been suggested that a missense substitution in the ZEB1 protein are associated with FECD and keratoconus [74].

### 3.3. MiRNAs of the Epithelial-to-Mesenchymal Transition in Fuchs’ Endothelial Corneal Dystrophy

#### 3.3.1. MiRNA and the Cornea

Recently, studies of miRNA profiles in healthy ocular tissues were performed, providing miRNA transcriptomes of different ocular tissues [75]. The most abundant miRNAs found in healthy eye tissues were miR-143-3p, miR-184, miR-26a-5p, and miR-204-5p [75].

Overall, in the cornea 297 miRNAs are expressed. Of them, 18 are specific [75], and 11 of those share 28 genes—10 regulated in the first place by the exclusively expressed miRNAs BAP1 (100%), DLX4 (100%), IL24 (100%), INPPL1 (100%), RERE (100%), SIP1 (100%), WASF3 (75%), ZEB1 (88%), ZEB2 (88%), and FPM 2 (100%).

For the epithelium of the cornea a distinct expression of miR-184 is characteristic [75,76,77,78].

For the corneal endothelium very little evidence can be found. miR-184, which, as said above, is highly expressed in the corneal epithelium, was primarily expressed also in the corneal endothelium of an adult mouse eye [76,79].

#### 3.3.2. Corneal Endothelium Senescence and miRNA

In the corneal endothelium of a mouse model 27 miRNAs were differently expressed in correlation to aging. In the samples of corneal endothelium of aged mice as opposed to the offspring, 20 miRNAs were downregulated, and this was less than 0.5-fold. Seven miRNAs were upregulated, and this was more than 1.5-fold. Critical miRNAs for regulating the aging of corneal endothelial cells of mice are considered to be miR-29c, miR-34c, miR-124, miR-695, and miR-32 [80]. MiR-695, miR-31, miR-190, miR-183, miR-182, and miR-194 are the most remarkably downregulated miRNAs, and miR-34c and miR-124 are the most remarkably upregulated. They were included in the signaling pathways of the glutamatergic-synapse pathway, the phosphatidylinositol-signaling pathway, the neurotrophin-signaling pathway, the TGF- β1-signaling pathway, and oxidative phosphorylation [80].

In the human endothelial cell line (hCEC), a booster role of miR-30c-1 in cell propagation was proven. It was found that cell-aging effects of TGF- β1 were converted by miR-30c-1, which rendered miR-30c-1 a feasible treatment molecule for hCECs regeneration [81].

The different miRNAs from the miR-30c family—miR-30c-1 and miR-30c-2—have specific nucleotides at the 3p and may thus also be disparate in their functional roles. The somewhat-controversial purpose of miR-30c-1 was proposed to be the promotion of cell-cycle progression. Therefore, the regulation of its activity may induce regeneration of hCECs [81,82,83].

miR-30c has been found to be decreased in expression in FECD [59].

#### 3.3.3. Corneal Endothelium miRNA and Oxidative Stress in Fuchs’ Endothelial Corneal Dystrophy

Oxidative stress is a well-known trigger of cell damage, and it was suggested to be an important aspect in FECD disease development. Ex vivo, oxidative stress caused characteristic morphological changes and apoptosis of corneal endothelial cells, which resembled clinical findings characteristic of FECD [84,85,86]. miRNA-34a is a p53-inducible miRNA, which in coordination with the increased c-Myc downregulates CD44 antigen expression [87]. In endothelial cells, it was proven that oxidative stress causes depressed miR-34a expression and elevated c-Myc. The expression of CD44 antigen was upregulated. The mitochondria metabolic homeostasis was impaired, which led to corneal endothelial-cell failure [88]. The levels of MiR-34a mimics were also connected to cellular pHi, which in turn influenced the transit of mitochondrial respiration to oxidative phosphorylation [89]. The repressed miR-34 followed by elevated CD44 antigen activated Ras homolog gene-family member A (Rho A) and matrix metalloprotease 2 (MMP-2).

#### 3.3.4. miRNA in Fuchs’ Endothelial Corneal Dystrophy

There were 311 of 754 tested (41.2%) miRNAs detectable in corneal endothelial cells both from FECD patients and normal patients. Almost 90% of all detected miRNAs in endothelial cells are downregulated in FECD, one third of them statistically significantly [59].

The underlying reasons for this universal miRNA downregulation in FECD, as opposed to un-diseased corneas, is downregulation in a gene, which is crucial for miRNA processing. Namely, a 1.38-fold (*p* = 0.039) downregulation of *DICER1* was demonstrated in FECD endothelium. The transcriptional expression of other similar miRNA-processing essential genes *DROSHA* (1.03-fold, *p* = 0.82) and *DGCR8* (DiGeorge syndrome critical region 8 gene) (1.11-fold, *p* = 0.60) were not changed remarkably [59].

Three miRNAs from the miR-29 family (miR-29a-3p, miR-29b-2-5p, and miR-29c-5p) were significantly downregulated. When FECD samples were compared to normal ones, miR-29a was classified as one of the three most deregulated miRNAs in FECD.

The members of the miR-29 family have many similar sequences and even identical seed regions, and that is why the diapason of their target genes overlaps to a large extent [90]. The miR-29 family plays an important role in the regulation of ECM turnover and fibrotic conditions of different organs and tissues, including the eye [90,91]. In FECD samples compared to normal ones, a significant transcriptional overexpression of two out of three experimentally validated mir-29 targets was found (mirWALKdatabase, http://www.umm.uni-heidelberg.de/apps/zmf/mirwalk/ (accessed on 1 July 2022)): (1) *COL1A1*—42.65-fold increase and (2) *COL4A1*—4.21-fold increase.

The third experimentally validated mir-29 target *LAMC1* (laminin subunit γ 1 gene) also showed a trend for transcriptional upregulation with 1.35-fold overexpression [92]. In samples of FECD corneas, distinct collections of collagen I and collagen IV under the corneal endothelium were proven, whereas laminin was collected in the endothelial cells without any extracellular deposits. [92].

Medical-based modifications of miRNA expression could be a non-invasive approach to patients with FECD. For example, the Rho-associated kinase inhibitor fasudil was proven to stop renal fibrosis and re-establish the expression of miR-29 [93]. This drug has a well-known favorable clinical effect in FECD, and the possible underlying similar miRNA mechanisms would be an interesting topic of further research [94].

#### 3.3.5. miRNA, Endothelial Mesenchymal Transition in Fuchs’ Endothelial Corneal Dystrophy

Mutation in the seed region of miR-184 was found in both endothelial dystrophy–iris hypoplasia–congenital cataract–stromal thinning syndrome (EDICT syndrome) and FECD [95]. Namely, a single-base substitution in the seed region of miR-184(+57C > T) was described in a syndrome of endothelial corneal dystrophy with iris hypoplasia, congenital cataract, and stromal thinning—so-called EDICT syndrome. Patients with this syndrome have a hazy cornea and corneal endothelial changes that resemble FECD. The endothelium has a beaten-metal appearance and histologically prominent posterior nodules with impaired function similar to the endothelium in FECD [95,96,97].

The miR-184(+57C > T) variant alters the DICER-binding or RISC assembly, which in turn influences the mature miR-184. Either the expression is reduced, the activity is decreased, or the mature miR-184 is completely changed [95]. The defective miR-184(+57C > T) variant cannot sufficiently compete with another miRNA, miR-205. The miR-205-related knockdown of the inositol polyphosphate phosphatase-like 1 gene (INPPL1) is thus avoided, and this leads to dysregulation of the protein kinase B (Akt)-signaling pathway and EMT [50,95,98].

EMT stands for a mechanism of transition of polarized cells to a migratory phenotype. The impaired EMT was proposed as a common mechanism in corneal endothelial disorders. The result is compromised reparation of central corneal endothelial cells, which involves moving the endothelial cell from the periphery to the center [50].

miRNA profiles of corneal endothelial cells in FECD were compared to normal corneas. The expression of miRNAs in general was decreased in FECD [59]. There were similar one-way changes in levels of miRNAs reported in other diseases [99,100,101,102]. The reasons for such unidirectional downregulation could be alternations in primary miRNA transcripts, in the generation of miRNA, in argonaut protein production, or in miRNA turnover [59].

MiR-29a is one of the three miRNAs of the same family that are the most downregulated mature miRNAs in FECD endothelium. Studies of miR-29 targets proved a consequent increase in expression of collagen I, collagen IV, and laminin in FECD samples. It was advocated that downregulation of miR-29 results in an aggregation of collagens and other ECM-linked components in the subendothelial space in FECD. The miRNAs from the miR-29 family are the main regulators of the ECM. Expression of these miRNAs result in impeded production of various ECM-related transcripts and proteins. The targets of individual miRNAs from the miR-29 family substantially overlap because all of them have several similar sequences and even identical seed regions [90].

Abnormal expression of miR-29 was reported in liver, kidney, heart, and lung fibrotic diseases [98,103,104,105,106,107]. Additionally, ECM was proven to be under the influence of miR-29 in different ocular tissues such as Tenon’s capsule or the trabecular meshwork [99,108,109].

An excessive expression of genes that bring on EMT, such as ZEB1 and SNAIL1, was proven in FECD corneal endothelial cells. The overexpression of these genes was suggested as the basis of elevated susceptibility of FECD corneal endothelial cells to TGF-β [65]. Stimulation of cells with TGF-β inhibited miR-29 in other tissues, resulting in expanded production of collagen I [108]. Analysis of the expression of EMT-related cytokines in the aqueous humor of eyes with FECD before and after cataract surgery proved an increase in TGF-β 1, TGF-β 2, and MCP-1 in eyes after cataract surgery. This finding was consistent with the theory of prolonged change in ocular microenvironment after natural-lens removal. Furthermore, a connection between aqueous-humor concentrations of TGF-β 1 and MCP-1 and thickening of the central cornea in FECD eyes after cataract surgery leads to the idea that increased levels of these cytokines might cause postoperative corneal decompensation [62]. However, the direct impact of TGF-β on miR-29 expression in corneal endothelial cells of FECD has not been studied yet.

## 4. Discussion

The pathophysiology of FECD was connected to different genetic and environmental aspects [1,36], the latter being in the first place connected to the constant presence of oxidative stress. The endothelium in FECD undergoes EMT with functional changes and pump-function loss, resulting in corneal edema and opacification [49].

The clinical picture in an early stage of FECD is difficult to differentiate from normal aging corneal changes. Thus, a biomarker of FECD would unambiguously prove the pathological process and support decisions regarding an early surgical intervention [6].

MicroRNAs were suggested to be suitable biomarkers of diseases, and they can be found in tissues and body fluids, both intra- and extracellular. The literature review of EMT biomarkers in Fuchs’ demonstrated significant miRNA-184 and miRNA-29 changes in diseased corneal tissue.

Almost a decade ago, Dunmire et al. suggested that miRNAs exist in the aqueous humor, both in solution associated with RNA-binding proteins and contained within extracellular vesicle exosomes [110]. Aqueous-humor samples can be obtained minimally invasively for analysis of biomarkers. The aqueous humor bathes the endothelium, where the initial changes of FECD occur, and could thus be a source of early clues to the disease of the corneal endothelium. The report by Dunmire et al. raised interest in further study of aqueous-humor RNA profiles and their use as ocular-disease biomarkers.

However, initial studies were not successful due to the small amount of total RNA in non-cellular samples of aqueous humor. The concentration of total RNA in the aqueous humor was proven to be 14–85 ng/mL [111], which resembles human breast milk at 9.7–228.2 ng/mL [112] and is minor to other body fluids [113]. It was undetectable with the usual spectrophotometric procedures. The following studies were performed with advanced RNA-isolation methods, mainly in search of biomarkers for glaucoma [111,114,115]. As far as aqueous-humor analysis of EMT in FECD proteome changes [116] and cytokines of EMT in FECD [62], no epigenetic studies have been conducted.

Mature miRNAs can be found in cells in RNA granules, endomembranes, and mitochondria, and are released to extracellular space in exosomes. The miRNAs in aqueous humor arise both from the cells of eye structures and from the blood [116]. The extracellular nanovesicles or exosomes are an essential component of aqueous humor in this regard [117]. In fact, exosomes carry proteins and RNAs—mRNA and small RNAs (e.g., miRNAs)—to adjacent receptor cells [118].

In the past, exosomes were recognized as a manner of elimination of cellular waste. However, a new role of conveying exosomal RNA between cells has been found, and thus poses a possible novelty role among biomarkers of human disease [119,120].

In different cancer studies, EMT was also proven to be controlled by other non-coding RNAs, especially by long non-coding RNAs (LncRNAs) [121,122,123]. The lncRNA-miRNA crosstalk was suggested as the underlying control of the multi-step process of EMT in tumor progression. miRNAs are considered to be central post-transcriptional gene regulators and lncRNAs the “transcriptional noise” [124]. Different lncRNAs were demonstrated to have a role in the cellular proliferation, apoptosis, and anti-oxidative stress ability of corneal endothelial cells [124], as well as in pathological processes as corneal neovascularization and inflammation [125,126]. There are, to date, no studies showing the role of lncRNAs in EMT of corneal endothelial cells or in connection to FECD and should be the question of further studies.

In order to have a biomarker for FECD, the specific miRNAs of EMT or even a specific lncRNA–miRNA crosstalk should be proven in aqueous humor, which is suggested for further studies.

## Data Availability

Not applicable.

## Abbreviations

AGBL1	ATP/GTP binding protein like 1
Akt	protein kinase B
COL_A_	collagen type _ α _chain
DGCR8	DiGeorge syndrome critical region 8 gene
DMEK	Descemet membrane endothelial keratoplasty
DSO	Descemet stripping only
DWEK	Descemet without endothelial keratoplasty
EDICT syndrome	endothelial dystrophy–iris hypoplasia–congenital cataract–stromal thinning syndrome
EMT	epithelial mesenchymal transition
FECD	Fuchs’ endothelial corneal dystrophy
FGF-2	fibroblast growth factor 2
hCEC	human endothelial cell line
IL-1β	interleukin 1 β
INPPL1	inositol polyphosphate phosphatase-like 1 gene
LAMC1	laminin subunit γ 1 gene
LncRNA	long non-coding RNA
LOXHD1	lipoxygenase homology domains 1
MCP-1	monocyte chemoattractant protein-1
miRNA	microRNA
MMP	matrix metalloprotease
mRNA	messenger RNA
PPCD	posterior polymorphous corneal dystrophy
Rho A	Ras homolog gene-family member A
RISC	induced silencing complex
SLC4A11	Solute-carrier family 4 sodium borate-transporter member 11
SNAI 1	Snail-family transcriptor repressor 1
TCF4	transcription factor 4
TCF4	transcription factor 4 gene
TCF8	transcription factor 8 gene
TGF-β	transforming growth factor–β
TNF-α	tumor necrosis factor α
UTR	untranslated region
ZEB1	Zinc-finger E-box-binding homeobox 1
